# On-call or not on-call, what difference does it make in paediatric radiology?

**DOI:** 10.1186/s13244-025-01948-0

**Published:** 2025-03-26

**Authors:** Willemijn M. Klein, Amaka C. Offiah, Ola Kvist, Karen Rosendahl

**Affiliations:** 1 Department of Medical Imaging, Radboudumc/Amalia Children’s Hospital, Nijmegen, The Netherlands; 2 Division of Clinical Medicine, University of Sheffield, Sheffield, UK; 3 Department of Radiology, Sheffield Children’s NHS Foundation Trust, Sheffield, UK; 4 Department of Radiology, Columbia University Medical Center, New York, NY USA; 5 Department of Women’s and Children’s Health, Karolinska Institute, Stockholm, Sweden; 6 Department of Radiology, University Hospital of North Norway, UiT the Arctic University of Norway, Tromsø, Norway; 7 Faculty of Health Sciences, Department of Clinical Medicine, UIT the Arctic University of Norway, Tromsø, Norway

**Keywords:** After-hours care, Child, Diagnostic errors, Radiology, Paediatrics

## Abstract

**Objectives:**

There is an ever-increasing demand for out-of-hours expert opinion in paediatric radiology, which cannot be delivered in all hospitals. This study was designed to ascertain whether paediatricians, paediatric surgeons and radiologists are satisfied with the current situation; and to investigate the extent to which diagnostic errors are made while on-call with either residents, general or paediatric radiologists reporting on paediatric examinations.

**Methods:**

Two surveys were compiled and dispatched. The first, is to paediatricians, paediatric surgeons and paediatric radiologists questioning their satisfaction with the current on-call paediatric radiology services in their hospitals. The second, is to paediatric radiologists inviting them to retrospectively score the accuracy of the reporting on consecutive paediatric radiology examinations performed during on-call hours in their hospitals.

**Results:**

The first survey revealed that 40/49 (82%) paediatric physicians were satisfied with the paediatric radiology service during office hours, decreasing to 33% during on-call hours. In the second survey, a total of 464 on-call paediatric radiology examinations were analysed, demonstrating 20.2% misdiagnoses. General radiologists had more misdiagnoses and were slower in providing a report than residents.

**Conclusion:**

The current service with a lack of on-call paediatric radiologists, is associated with increased misdiagnoses and dissatisfaction among physicians and requires improvement.

**Critical relevance statement:**

This study shows that it may be a struggle to organise the 24-h availability of an expert paediatric radiologist, yet this might avoid 20% of misdiagnoses, half of which have direct clinical consequences.

**Key Points:**

- The current organisation of paediatric radiology on-call rotas is unsatisfactory for many clinicians.
- A substantial amount of on-call paediatric radiology reports contain misdiagnoses, and these may have significant clinical consequences.
- Hospitals should reconfigure out-of-hours paediatric radiology covers.

**Graphical Abstract:**

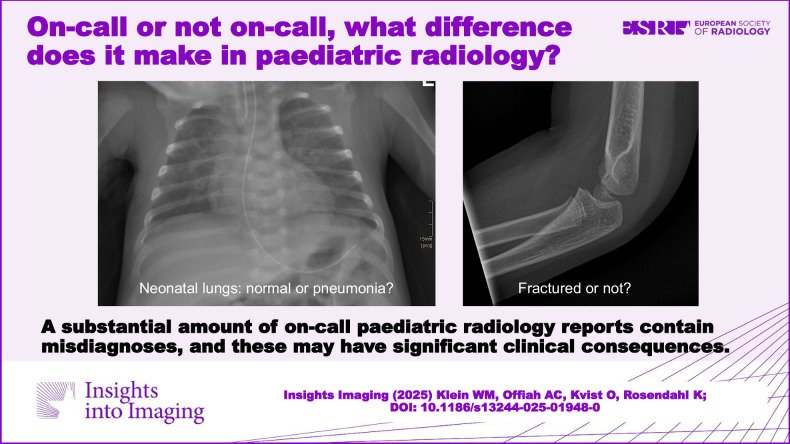

## Introduction

There is a growing demand for 24-h expert paediatric radiology services [1]. Currently, only around 25–50% of imaging studies that are performed in children are reported by radiologists (or radiographers) with subspecialty training in paediatric radiology, and most centres (62% in the United Kingdom, 90% in Norway) do not have access to 24-h paediatric radiology expertise [2, 3]. Across Europe, many paediatric examinations are therefore reported by general or non-paediatric radiologists, residents, or clinicians; exact numbers are not available. It has come to the attention of the authors, that paediatricians, both in general and academic hospitals, have an increasing demand for expert paediatric radiology services during on-call hours^*^ (^*^on-call hours: the time outside regular office hours, generally weekdays from 6 p.m. to 8 a.m., weekends, and holidays). The shift from daytime paediatric radiologists’ expertise to on-call cover by general (non-paediatric) radiologists and residents, is becoming less acceptable due to the concern of potentially delayed or incorrect diagnoses and possible adverse consequences for the patient.

The increasing demand for a 24-h expert radiology opinion is challenging, particularly for medium-sized and small paediatric units, as the number of cases in need of an acute paediatric radiology opinion and the number of trained paediatric radiologists, are limited [4, 5]. More paediatric radiologists working during on-call hours means fewer of them present during the day [6]. Therefore, most hospitals are reluctant to organise a separate paediatric on-call rota. The question is whether we should reconsider the current situation. As the population expands, and the need for more complex radiological investigations becomes more widespread, there will be increased demand for skilled interpretation of paediatric imaging [7]. In fact, whilst paediatric healthcare has improved over the last 10 years, with a significant reduction in mortality, the number of emergency hospital admissions amongst babies and children < 4 years has increased by 30% and 28%, respectively [8], similar to the 28% increase in the previous decade (1999–2010) [9].

In centres without specialist paediatric services, paediatric radiologists often participate in general on-call rotas, meaning that specialist knowledge is provided on an ad-hoc basis and non-paediatric radiologists and residents are required to take up their remaining caseloads and provide a paediatric opinion. Some centres that cannot provide on-call hours paediatric expertise, now refer children to a specialist centre, which is likely to improve outcomes for the children concerned. Incomplete paediatric radiology cover may lead to a significantly higher major error rate in general compared to specialist paediatric hospitals, as was the reported situation in a centre in the United States of America [10].

The aim of the study was two-fold. First, to examine the level of (dis)satisfaction of paediatricians, paediatric surgeons and paediatric radiologists with current radiology on-call services. Second, to examine the accuracy of diagnosis provided during on-call hours.

## Methods

Two surveys were distributed via the newsletters of the European Societies (Supplementary Material 1). The first, a questionnaire, was sent to paediatric radiologists, paediatric surgeons and paediatricians in Europe via their respective societies (European Society of Paediatric Radiology, Gesellschaft für Pädiatrische Radiologie, European Paediatric Surgeons’ Association, European Young Paediatricians’ Association and European Paediatric Association) between September 2022 and August 2023. The questionnaire was designed and piloted by W.M.K. and K.R. (each with over 20 years of experience in paediatric radiology) and included 15 questions covering: (1) existing paediatric radiology on-call-systems, (2) their satisfaction with the radiological services provided by both in and out of hours on a 5-point Likert scale, (3) their preferred level of service, (4) an inventory of typically well-diagnosed diseases/cases and misdiagnosed cases seen during on-call hours and (5) potential solutions for improvement. We calculated the shift of satisfaction by subtracting the Likert scale points from in to out of hours.

In a second survey, the members of the European Society of Paediatric Radiology were invited to collect data on 100 consecutive cases during paediatric radiology on-call hours. The invitations were submitted via the European Society of Paediatric Radiology e-newsletters from September 2022 to August 2023. Cases were required to be at least a year older than the date of the start of this review, in order to omit any remaining clinical consequences. In addition to age, sex, time of examination and interval between request and examination, data included: (1) radiological modality, (2) who provided and authorised the initial report (resident, non-paediatric or paediatric radiologist), (3) initial diagnosis, (4) retrospective expert opinion by the local paediatric radiologist who responded to the survey (named as the ‘responding paediatric radiologist’) and (5) if the new diagnosis was a clinically relevant change, possibly altering the patient’s treatment plan or clinical outcome. The responding paediatric radiologists collected data on consecutive cases from their hospital electronic patient files and therefore were not blinded to any information regarding the patient or first reporter.

Due to diverse radiology training and working situations across European countries, we provided the following definitions to the survey responders. A paediatric radiologist was defined as a radiologist with at least 1 year of working experience in a paediatric hospital. All other radiologists, including fellows, were defined as non-paediatric or general radiologists, without further specifications of additional expertise, and in this paper referred to as ‘general’ radiologists. A ‘resident’ was defined as a radiologist in training with various degrees of experience and subspecialty interests.

### Statistical analysis

Data were registered and analysed in IBM SPSS Statistics for Windows, version 29.0 (Armonk, NY: IBM Corp). Descriptive statistics, reporting numbers and percentages were calculated. Statistical differences were calculated, using suitable tests according to the data and distribution (*t*-test, ANOVA), with statistical significance set on *p*-values lower than 0.05.

## Results

### Survey

A total of 49 colleagues with varying European affiliations answered the survey; 14 paediatric surgeons, 17 paediatricians and 18 radiologists, of whom 12 were paediatric radiologists. (Supplementary Material 2) One paediatric surgeon had the same affiliation as one of the paediatric radiologists, all other respondents worked at different institutions.

There were 27 respondents working in university hospitals and according to 25 (92%) of them, paediatric examinations were reported by paediatric radiologists during office hours, decreasing to five (18.5%) during on-call. Corresponding figures for non-university teaching, non-teaching and private hospitals were five of 22 respondents (22.7%) during office hours, significantly lower than in the university hospitals, and four (18.1%) during on-call hours, comparable to the university hospitals.

On the question addressing satisfaction during office hours, 14/14 (100%) of paediatric surgeons, 10/17 (58.8%) paediatricians, 12/12 (100%) paediatric radiologists and 4/6 (66.7%) non-paediatric radiologists were satisfied or more than satisfied with their current situation. The only *dissatisfied* paediatricians (five in total, 5/17 (29.4%)) all worked in non-university hospitals without the availability of a paediatric radiologist both during office and on-call hours. During the on-call hours, the corresponding figures for satisfied or very satisfied were 6/14 (42.9%) paediatric surgeons, 2/17 (11.8%) paediatricians, 6/12 (50%) paediatric radiologists and 2/6 (33.3%) general radiologists, respectively (Supplementary Material 2).

The mean shift in the Likert satisfaction scale from office to on-call hours was −1.1 for paediatric radiologists, −0.7 for general radiologists, −0.88 for paediatricians and −1.2 for paediatric surgeons. The mean shift in satisfaction was −1.2 in university hospitals, −0.6 in non-university teaching hospitals, −1.2 in non-teaching hospitals and 0 in private hospitals. There were no statistically significant differences.

To the question, “Who would you prefer to provide on-call paediatric radiology service?”, 40 of 49 (82%) answered a paediatric radiologist, either for all paediatric cases (*n* = 14), for all children up to 4 years of age (*n* = 9), for all hospitalised children (*n* = 8), for all intensive care children (*n* = 4) or case-based whenever an expert is advantageous (*n* = *5*).

Yet three respondents answered that for paediatric cases, an on-call resident suffices, and six respondents felt that a non-paediatric or a general radiologist would suffice. Four of these nine responders added that there should be specific training in paediatric radiology, or a paediatric radiologist should be available to help in complex cases. These nine responders included one paediatric surgeon, two paediatric radiologists from university hospitals, and two general radiologists and four paediatricians from non-university teaching hospitals.

Suggestions on how to improve the existing paediatric radiology on-call system were given by 27 of 49 (55%) respondents in an open text field. Suggestions included improving the training of both paediatric radiology residents and general radiologists, or having a consultant paediatric radiologist in general hospitals. Other suggestions were to make use of teleradiology with a regional, national or even international network of on-call paediatric radiologists. Flexible working hours to cover the out-of-office duties were suggested by one paediatric radiologist. Others pointed out that the most difficult aspect of paediatric radiology consists of hands-on ultrasonography, which cannot be performed remotely. Further comments regarding additional costs, limited capacity and too heavy a workload on paediatric radiologists, were also submitted.

There was a wide variety of ailments that the responders felt were well diagnosed during on-call hours, and the same variety for the typical misdiagnoses, as shown in Supplementary Material 2. Intussusceptions, malrotation, hypertrophic pylorus, appendicitis, and pneumonia were all mentioned as both typically well-diagnosed by some respondents, and as typically misdiagnosed by others. Paediatric radiologists mentioned the acute abdomen and intussusception to be well diagnosed during on-call hours (the latter by ultrasound). Diagnoses that they considered more problematic to make during on-call, were the radiographs from the neonatal intensive care unit, as well as cerebral imaging. The opinions of the paediatric surgeons varied: some felt that intussusception and volvulus were typically well diagnosed during on-call hours whereas the paediatric surgeons that were dissatisfied with the on-call service in their hospital mention the same diagnoses as typically misdiagnosed. Both the non-paediatric/general radiologists and the paediatricians reported that cerebral magnetic resonance imaging is generally challenging to interpret.

### Case survey

Seven European paediatric radiologists, all from different centres and countries in North, West and Southern Europe, responded to the survey and reported on a total of 464 paediatric admissions (203, 43.8% girls). The children were 0 to 17 years of age, with a median age of 6.0 years. Most cases (401; 86.4%) were seen in university hospitals; the remaining 63 (13.6%) in non-teaching hospitals. Just under half (207, 44.7%) of the cases presented during weekends, between Friday evening and Monday morning. Of the 464 examinations, there were 204 (44.0%) conventional radiographs, 110 (23.7%) computed tomography examinations, 99 (21.3%) ultrasound examinations, 42 (9.1%) magnetic resonance scans and 9 (1.9%) fluoroscopies.

**Fig. 1:**
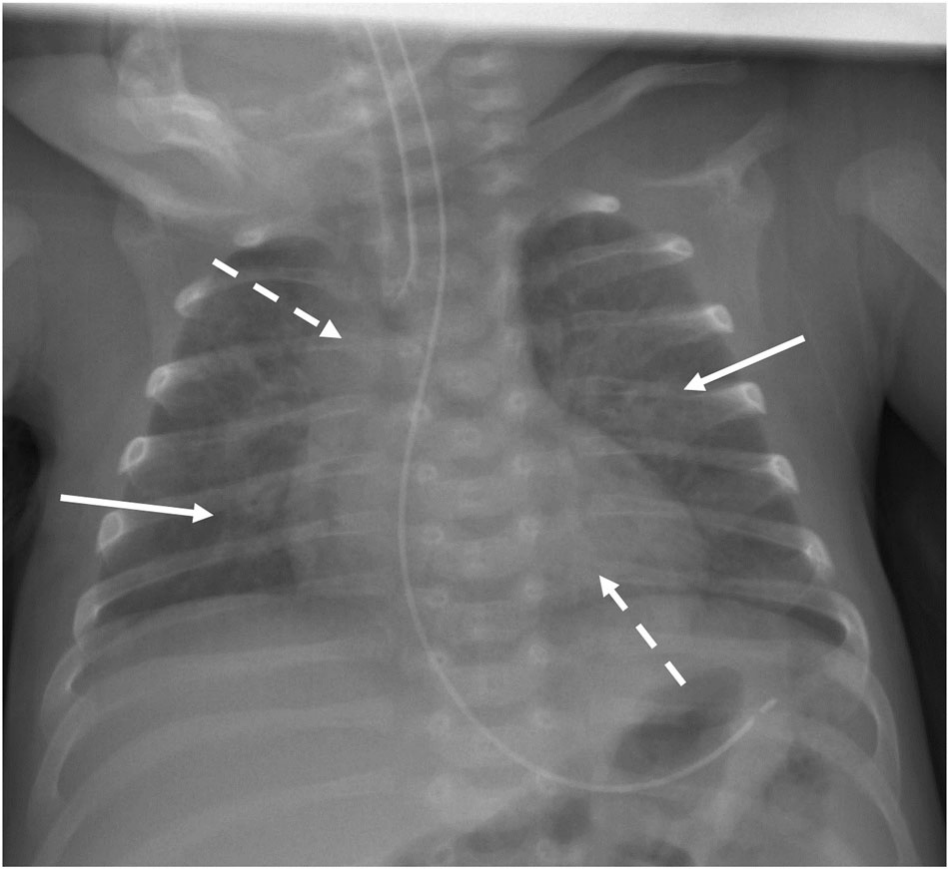
A female baby born at 28 weeks gestational age developed apnoeas 8 weeks after birth. Among other tests, the neonatologist requested a chest radiograph. The resident on call reported normal findings. This was corrected by the paediatric radiologist, who diagnosed infectious pulmonary disease, based on the bronchial wall thickening (arrows) and patchy consolidations (broken arrows)

**Fig. 2:**
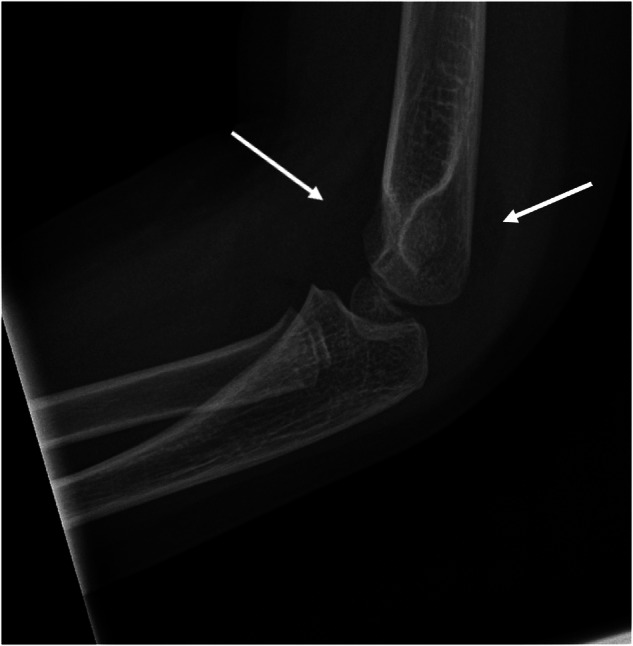
A three-year-old girl with a painful elbow after a fall. The first reporter missed the positive anterior and posterior fat pad sign (arrows) and the subsequent diagnosis of an elbow fracture

Residents were the first reporters in 282 cases, of which 233 (82.6%) diagnoses were later confirmed by the responding paediatric radiologist in the setting of this retrospective survey. Of the 49 (17.4%) cases that were disputed by the paediatric radiologist, 25 (8.9%) were considered to have a direct clinical consequence, if correctly diagnosed at the time of the requested radiological test, considering the medical circumstances of the child. (Figs. 1 and 2) General (non-paediatric) radiologists reported 123 cases, with 45 (36.6%) being challenged, of which 29 (23.6%) had direct clinical consequences according to the responding paediatric radiologist. The responding paediatric radiologist agreed with all 59 reports by paediatric radiologists. Chi-square showed that these discrepancies were statistically significant (χ^2^ 37.1 (*n* = 464), *p* < 0.001).

In all 94 of 464 (20.3%) cases in which the responding paediatric radiologist disagreed with the first reporter, a new diagnosis was given. A detailed list of the first reporter’s diagnosis as compared to the retrospective diagnosis by the responding paediatric radiologists is given in Table 1, demonstrating a diversity of diagnoses. The locations of the disagreements were in the brain (*n* = 17), chest (*n* = 21), abdomen (*n* = 15), musculoskeletal (*n* = 37) and further subcutis (1), vessels (1), drains and catheters and imaging technique (2). There were no associations between agreement/disagreement and the sex and age of the patient or the time interval between request and examination. All 94 cases of misdiagnoses were considered clinically important, either for therapeutic, prognostic or informative reasons. Yet in hindsight, the responding radiologist considered that in 54 (57%) cases, the new findings would have had immediate therapeutic consequences, had the diagnosis been correctly made at the time of imaging. These included missed or misdiagnosed hypoxic-ischaemic injury, pneumonia, necrotizing enterocolitis, and fractures. The remaining 40 (43%) cases were considered to receive the appropriate therapy, despite the misdiagnosis.

**Table 1:** Original (first) paediatric radiology diagnosis by the first reporter during on-call hours, together with the new, retrospective diagnosis of the respondent paediatric radiologist

First diagnosis	New diagnosis
Neuro	
Normal	Severe hypoxic-ischaemic injury and subdural haemorrhage
Leptomeningitis	Leptomeningeal seeding metastases
Acute ischemia	Neurometabolic crisis (probably mitochondrial disease)
Unremarkable	Pituitary macroadenoma
Unremarkable	Hyperammonemia bilateral basal ganglia involvement
Brain mass	Brain parenchymal haematoma and intraventricular haemorrhage
Encephalitis	Posterior reversible encephalopathy syndrome (PRES)
Demyelinating disease	PRES
Unremarkable	Polymicrogyria
Demyelinating disease	Leukodystrophy
Encephalitis	Hypoxic-ischaemic injury of a term neonate
Unremarkable	Takayasu arteritis
Cephalohematoma + subarachnoid haemorrhage (SAH)	Cephalohematoma, no SAH
Unremarkable	Symmetrical deep hypoxic injury
Unremarkable	Ectopic posterior pituitary
Pituitary microadenoma	Pars intermedia cyst
Parotitis with subcutaneous oedema	Subcutaneous inflammation without parotitis
Normal	Small birth-related subdural haemorrhage
Cardiothoracic	
Normal	Pneumonia
Regress pneumonia	Pneumatocele, thickened pleura, lymphadenopathy
Progressive consolidations	Bronchopulmonary dysplasia unchanged
Viral pneumonia	Pulmonary oedema secondary to myocarditis
Pulmonary arterial dilatation, pulmonary oedema	Pulmonary arteriovenous malformation
Normal	Pneumothorax
Respiratory distress syndrome (RDS)	No RDS but pneumonia
Normal lungs	Pulmonary oedema
RDS	No RDS but pneumonia
Normal	Pneumonia not mentioned
Normal	Pneumonia
Normal	Pneumonia
Normal	Severe pneumonia
Normal	Atelectasis
Lung cyst/congenital emphysema	Pneumomediastinum
RDS	Bronchopulmonary dysplasia (BPD)
Normal	Prominent pulmonary vessels
Pneumonia	Pneumonia and minor pleural effusion
Pleural effusion	No pleural effusion
Pneumonia	RDS with migrating opacifications
Abdomen	
Normal	Corpus alienum in the stomach
Constipation	Necrotizing enterocolitis (NEC) with perforation
Unremarkable	Focal pancreatitis
Unremarkable	Grade 2 liver injury
Unremarkable	Acute appendicitis
Magnets without bowel wall involvement	Likely the bowel wall between magnets
Peritonitis	Pancreatitis
Hydronephrosis	Hydronephrosis secondary to distal ureteral stone
Normal	Distal obstruction (intussusception)
No laceration spleen	Possible spleen laceration
Normal, no acute appendicitis	Adnexial cyst
Active Crohn’s disease	Active Crohn’s disease and ileoileal fistula
Kidney stone	Kidney and ureteral stone
Hydronephrosis	Ureteral stone
Musculoskeletal	
Fracture	No fracture
Hip arthritis	Transient synovitis of the hip
Normal	Septic arthritis
Unremarkable	Psoas hematoma in haemophilia patient
Possible stress fracture in talar bone (oedema)	Lateral hindfoot impingement
Normal	Fracture
Normal	Fracture
Normal	Fracture
Normal	Fracture
Radius fracture	Radius fracture with displaced epiphysis
Normal	Fracture
Normal	Fracture
Normal	Fracture
Fracture	No fracture
Normal	Fracture
Normal	Fracture
Normal	Suprapatellar effusion
Normal	Fracture
Normal	Fracture
Avulsion	Avulsion and fracture
Fracture	Normal ossification centres
Normal	Additional torus fracture
Normal	Non-ossifying fibroma
Radius fracture	Correction of the angular misalignment of the radius plus an ulnar torus fracture
Salter-Harris 2 fracture	Salter-Harris 4 fracture
Maybe fracture	Fracture
Suspected fracture	Normal variant
Fracture	Fractures with displacement and angulation
Suspected fracture	Fracture
Fracture	Multifragmental fracture
Suspected fracture	Fracture
Fracture	Additional fracture
Fracture	Shortened and displaced fracture
Positive fatpad	Effusion and fracture
Osteochondral defect	Osteochondral defect and a patellar dislocation
Minor avulsion	Locked joint
Normal	Effusion
General/multi-organ	
Dilated bowel	Pneumonia
Partial radiograph description	Additional description of thorax abdomen and lines
Line positions	Additional conclusion if lines are correct or malpositioned
Splenic laceration and thoracic compression fractures	Normal
SAH, dental fractures, and thoracic aortic dissection flap	No SAH, mandibular fractures, and no dissection

*PRES* posterior reversible encephalopathy syndrome, *SAH* subarachnoid haemorrhage, *RDS* respiratory distress syndrome, *BPD* bronchopulmonary dysplasia, *NEC* necrotizing enterocolitis

The time interval between a radiology request and imaging and from imaging to report, as well as the overall time from request to report, differed depending on the first reporter. Residents were the fastest to initiate imaging after a request was made; paediatric radiologists were the swiftest to report. Overall, residents were the fastest workers, with the non-parametric ANOVA (Kruskal–Wallis) test proving this to be statistically significant (*p* = 0.004). General radiologists were slower to report on the imaging than both residents and paediatric radiologists (Table 2).

**Table 2:** Speed of paediatric radiology per type of the first reporter

	Time interval		
First reporter	Request to imaging (median, range) (min)	Imaging to report (median, range) (min)	Request to report (median, range) (min)
Paediatric radiologist (*n* = 58)	56.5 (1261)	21.5 (960)	114 (1312)
General radiologist (*n* = 112)	86.0 (799)	63.5 (1276)	144 (1397)
Resident (*n* = 260)	43 (1190)	34.0 (879)	100.5 (1208)
Kruskal–Wallis H (d*f*), *p*-value	16.4 (2) < 0.001	21.9 (2) < 0.001	11.0 (2) 0.004

The resident is significantly faster than both the general and paediatric radiologist to work from request to report. The general radiologist was significantly slower than the paediatric radiologist to report after imaging

## Discussion

Our results indicate that only one out of four (26%) paediatric surgeons and paediatricians are satisfied with the existing, or rather non-existent, paediatric radiology on-call hours service, particularly for more complex cases. Moreover, around one in five of the diagnoses provided by a resident or general/non-paediatric radiologist on-call was suboptimal or even inaccurate, retrospectively, which would probably have led to change in therapy, prognosis, and/or patient information given.

Our study shows that the limited availability of paediatric radiologists during on-call hours compared to regular office hours is a significant source of concern for both paediatric surgeons and paediatricians. This issue is evident not only in non-teaching hospitals but also in 80% of university teaching hospitals, where initial radiology reports during on-call periods are frequently provided by general radiologists or residents without specialised paediatric radiology training. However, in six out of thirteen university hospitals, support from a paediatric radiologist was available for specific indications, suggesting that this model may serve as a potential solution. A promising approach could involve the implementation of a tertiary on-call service, where the overall workload of paediatric radiologists is carefully managed, but their expertise is accessible for complex cases. Our case review highlights that these difficult cases spanned a range of conditions, including fractures, pneumonia, and cerebral vasculitis. Based on this, we argue that the availability of a paediatric radiologist should be guaranteed for all paediatric cases, not just those deemed complex. A possible solution would be to integrate paediatric radiologists into an existing regional network of hospitals, as demonstrated by certain metropolitan areas in the United States, where a paediatric hospital collaborates with other specialised and general hospitals. Efficiency could be improved by virtually or digitally centralising paediatric radiology expertise [11]. Broader national or international paediatric radiology networks could be feasible, though the challenges of information-sharing, communication technology, and financial, and legal structures must be considered.

Several factors, however, might influence the successful establishment of a comprehensive 24-h paediatric radiology service. One critical issue is the shortage of paediatric radiologists, which limits the number of specialists available to participate in on-call rotations [4, 5, 7]. This shortage is exacerbated by a relatively low interest in paediatric radiology fellowships, further restricting the workforce [6]. Moreover, many general radiologists, including those with some paediatric radiology experience gained through focused internships during residency or fellowship, may not have the level of training necessary to handle complex paediatric cases autonomously. The insufficient training of residents and general radiologists in paediatric-specific imaging further complicates their ability to provide accurate and timely diagnoses during on-call hours. Addressing these gaps in education and skill development is essential to ensuring the success of any 24-h paediatric radiology service.

In addition to workforce and training challenges, logistic factors such as scheduling and financial considerations will also impact the viability of such a dedicated service. Paediatric radiologists often face high workloads, thus an additional on-call shift might lead to burnout if not carefully managed. Financial implications, including adequate compensation for on-call work and the potential need to hire additional staff, must be factored into the planning process. Furthermore, the costs of developing and maintaining the necessary communication technologies for effective regional or national collaborations should not be underestimated. Ensuring the sustainability of a paediatric radiology service will require careful balancing of these financial, scheduling, and educational considerations.

To the best of our knowledge, this is the first study to address user satisfaction and performance metrics for paediatric radiology services amongst our non-radiology colleagues. Previous studies have shown that discrepancy rates for second interpretations in studies of paediatric patients transferred to tertiary care paediatric institutions are substantial [10]. Although the original and second interpretations in most cases concur, some major discrepancies were prevalent—12.6% and 32.6% of neuroimaging and body studies, respectively—and the second interpretations were significantly correlated with the final diagnosis [10].

Moreover, the question of whether specialist expertise in paediatric radiology adds value, was addressed in a recent mini-symposium in the journal *Paediatric Radiology*. Opinions on the need for subspecialists in paediatric gastrointestinal and hepatobiliary radiology [12], on providing second-opinion interpretations of paediatric imaging, embracing the call for value-added medicine [13] and on the sustainability of paediatric radiology in Italy [14], were included amongst other themes. The authors highlighted the need for paediatric-specific approaches including good communication, in-depth knowledge of paediatric pathology and growth physiology, as well as flexibility and creativity to perform the best imaging with reasonably low disadvantages of ionising radiation, invasiveness and anaesthesia.

We acknowledge the limitations of this study. First, the number of respondents in the two surveys was low, and therefore there is likely to be selection bias. This may reflect the unawareness of the subject amongst the majority of the surveyed clinicians. Since the newsletters of the European Societies were used to spread the surveys, and not social media, this may have caused a prejudiced selection of respondents, for example, no responses were received from any Eastern European countries. Secondly, the retrospective nature of the case study may have given the responding paediatric radiologists the advantage of follow-up information and hindsight bias. Perhaps, despite having asked to use consecutive cases, one may have chosen a specific selection of the more interesting cases; this could not be verified by the authors and we trusted the responders’ integrity. Thirdly, the final diagnosis of the included cases was not confirmed. We recommend that future studies prospectively collect case data from more centres worldwide, to improve comparability to daily practice. We also advise, the implementation of a cost-effectiveness analysis to include delay in diagnosis, any additional imaging required and prolonged hospital stay.

## Conclusion

The current situation of not having an on-call paediatric radiologist in most European hospitals is suboptimal, leading to many misdiagnoses and unsatisfied care providers. This requires improvement.

## Supplementary information

ELECTRONIC SUPPLEMENTARY MATERIAL

## Data Availability

Data from the surveys are freely available upon reasonable request.
