# Supplementary material for: On-call or not on-call, what difference does it make in paediatric radiology?
Source: Insights Imaging. 2025 Mar 26;16:73. doi: 10.1186/s13244-025-01948-0 (PMC11947353; doi:10.1186/s13244-025-01948-0)
Supplement: Supplementary file 1 — ELECTRONIC SUPPLEMENTARY MATERIAL [file 13244_2025_1948_MOESM1_ESM.pdf]

On-call or not on-call, what difference does it make in paediatric radiology?

ELECTRONIC SUPPLEMENTARY MATERIAL

Supplementary Material 1.

Surveys on pediatric radiology on-call service

This survey is to measure quality and satisfaction of the pediatric radiology service in the on-call hours. Participation is voluntary and anonymized.

Survey 1. Pediatric radiology service

1. Which type of hospital do you work in
   a. University hospital
   b. non-university teaching hospital (any interns or trainees)
   c. non-teaching hospital (no interns or trainees)
   d. private hospital
   e. other, please specify:

2. What is your job
   a. Pediatric surgeon
   b. Pediatrician
   c. Pediatric radiologist*
   d. General / non-pediatric radiologist
   e. Other, please specify:

3. In which country do you work?
   a. Scroll menu ….

**please report on 100 consecutive pediatric radiology cases in the sheet [link]*

4. In your hospital, who reports (validates, supervises) pediatric radiology during routine daily hours:
   a. Resident
   b. General / non-pediatric radiologist
   c. pediatric radiologist
   d. other, please specify:

5. In your hospital, who reports (validates, supervises) pediatric radiology in the on-call hours?
   a. Resident
   b. general / non-pediatric radiologist
   c. pediatric radiologist
   d. other, please specify:

6. How satisfied are you with the ped rad reporting in the routine daily hours in your hospital?
   a. very unhappy

b. unhappy
c. neutral
d. happy
e. very happy

7. How satisfied are you with the ped rad reporting in the on-call hours in your hospital?
    a. very unhappy
    b. unhappy
    c. neutral
    d. happy
    e. very happy

8. Typical diagnoses that are generally well diagnosed and without delay:
    ….

9. Typical diagnoses that are at risk of being misinterpreted or cause delay before you receive a final report:
    …..

Potential actions for improvement:

10. Who would you prefer to report on pediatric radiology, in order to have good quality patient care?
    a. Resident
    b. General / non-pediatric radiologist
    c. pediatric radiologist
    d. other, please specify:

11. Would you prefer to have reports by a pediatric radiologist during the on-call hours?
    a. No, the on-call reports of non-pediatric radiologists suffices.
    b. Yes, for all pediatric radiology under age 16 yrs
    c. Yes, for all pediatric radiology under age 4 yrs
    d. Yes, for all radiology of hospitalized children
    e. Yes, for all radiology of the pediatric intensive care units.
    f. Other, please specify:

12. Would that mean additional costs? (extra personnel, financial reimbursement, travel costs, etc)
    a. Yes
    b. No
    c. I don’t know

13. Are financial resources available?
    a. Yes
    b. No
    c. I don’t know

14. Would a national or regional pediatric radiology on-call system be possible as far as information technology (IT) solutions are available?
    a. Yes
    b. No
    c. I don’t know

15. Do you have any specific advice for how to improve a pediatric on-call system?

…….

Survey 2.

Reporting pediatric radiology:

**Please report on 100 cases of pediatric radiology in the on-call hours / days. (web-based sheet)*

-type of hospital:

University hospital

non-university teaching hospital

non-teaching hospital.

- modality

CR

CT

MRI

US

radiography

other (specify);

- day of the week;

holiday yes/ no;

time of the imaging;

time of the report validation.

- first reporter is
A resident
general radiologist
pediatric radiologist
other, specify;

- Judgment on the original report:
agree
disagree without clinical consequence (plus mention the new finding)
disagree with clinical consequence (plus new finding);

- was this imaging involved in
a (parental) complaint
complication
calamity (with medical inspection)

Supplementary Material 2. Satisfaction with the paediatric radiology service during office and on-call hours. Likert scale -2 (very unhappy) -1 (unhappy) 0 (neutral) 1 (happy) 2 (very happy).

**Job**	**Hospital**	**Satisfaction with paediatric radiology service**											**n**
		**Office hours**						**On-call hours**					
		**-2**	**-1**	**0**	**1**	**2**		**-2**	**-1**	**0**	**1**	**2**	
**Paediatric radiologist**	University				5	4			3	2	3	1	
	Non-univ. teaching				1						1		
	Non-teaching				1				1				12
	Private					1						1	
**General radiologist**	University			1					1				
	Non-univ. teaching				3					1	2		
	Non-teaching				1				1				
	Private			1						1			6
**Paediatrician**	University			1	1	2			1	2	1		
	Non-univ. teaching		3	1	6			1	3	5	1		
	Non-teaching		2		1			1	1	1			17
	private												
**Paediatric surgeon**	University				4	9			5	2	1	5	
	Non-univ. teaching				1					1			
	Non-teaching												14
	Private												
Total		0 (0%)	5 (10%)	4 (8%)	24 (49%)	16 (39%)		2 (4%)	16 (33%)	15 (31%)	9 (18%)	7 (14%)	49 (100%)
